# Progress on Designing a Chemical Retinal Prosthesis

**DOI:** 10.3389/fncel.2022.898865

**Published:** 2022-06-10

**Authors:** Jiajia Wu, Corey M. Rountree, Sai-Siva Kare, Pradeep Kumar Ramkumar, John D. Finan, John B. Troy

**Affiliations:** ^1^Department of Biomedical Engineering, Robert R. McCormick School of Engineering and Applied Science, Northwestern University, Evanston, IL, United States; ^2^Department of Mechanical and Industrial Engineering, College of Engineering, University of Illinois at Chicago, Chicago, IL, United States

**Keywords:** retinal prosthesis, retinal degeneration, vision restoration, neurotransmitter-based prosthesis, glutamate stimulation, retinitis pigmentosa

## Abstract

The last major review of progress toward a chemical retinal prosthesis was a decade ago. Many important advancements have been made since then with the aim of producing an implantable device for animal testing. We review that work here discussing the potential advantages a chemical retinal prosthesis may possess, the spatial and temporal resolutions it might provide, the materials from which an implant might be constructed and its likely effectiveness in stimulating the retina in a natural fashion. Consideration is also given to implant biocompatibility, excitotoxicity of dispensed glutamate and known changes to photoreceptor degenerate retinas.

## Introduction

A decade has passed since the last review of chemical retinal prostheses (Iezzi and Finlayson, 2011), which focused on preliminary ideas and measurements critical to optimizing device design. Over the intervening period some of the ideas contained in that review article have been explored and it is now timely to examine progress made with an eye to the next stage of development. Recently, Strickland and Harris (2022) have drawn attention to some business and ethical challenges faced in bringing about the clinical application of retinal prostheses. Chemical retinal protheses remain distant from clinical application at this time, so these broader societal questions are not considered here. For the reader interested in such questions, Troy (2015) might be a good starting point.

Like all retinal prostheses, those using chemical stimulation, target a patient population with a largely intact retinal output. Generally, the patient population will be those subject to diseases of photoreceptor loss, such as those with retinitis pigmentosa or those with age-related macular degeneration, although there are those with photoreceptor loss through injury who could also benefit from retinal prostheses.

## Advantages of a Chemical Prosthesis

The key potential advantage chemical stimulation of the retina has over electrical stimulation is the fact that the natural activators of neurons are transmitter molecules; i.e., a chemical stimulus is more naturalistic. For both chemical and electrical stimulation, fine spatial vision requires close packing of stimulation sites. In the case of electrical stimulation, this necessitates small electrode tips with consequent high charge densities for neural stimulation resulting potentially in electrode tip erosion and the generation of chemical entities toxic to cells (Zheng et al., 2021). A chemical prosthesis with a high-density array of injection ports is itself challenging from an engineering perspective but, unlike with electrical prostheses, there does not seem to be a significant theoretical physical limit.

Another potential advantage of a chemical prosthesis might be its capacity to match itself to retinal circuitry. The most obvious example of this would be exploitation of the part played by glutamate release from photoreceptors in differentially activating OFF and ON bipolar cells (Whitaker et al., 2021), respectively, through their sign-conserving kainate or AMPA (DeVries, 2000) and sign-inverting mGluR6 (Pinto et al., 2007) receptors. But, additionally, the ability to release multiple chemicals (e.g., glutamate, GABA, glycine, acetylcholine, dopamine, kainate, AMPA) provides a wide set of neuromodulation possibilities. To date, effort has focused on injection of glutamate, the primary mechanism of excitatory neurotransmission from photoreceptors to bipolar cells to retinal ganglion cells. In this review article we consider only progress made toward a chemical prosthesis that dispenses glutamate.

## Components of a Chemical Retinal Prosthesis

**FIGURE 1:**
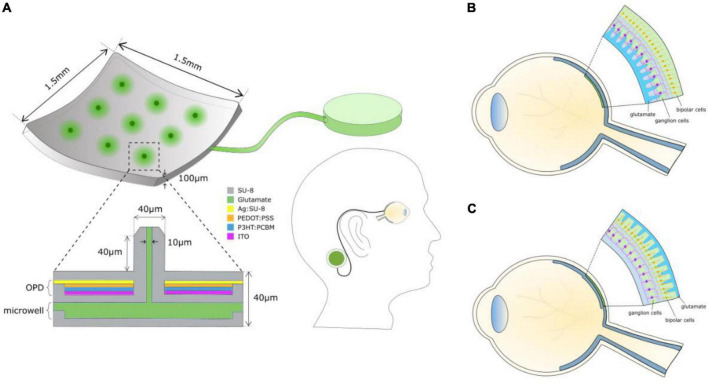
**(A)** Schematic of the components of a chemical retinal prosthesis for implantation. Envisaged is an implantable chip containing an array of glutamate injection microneedles, each fed from an intraocular well of glutamate, which is itself connected to an external large reservoir of glutamate, perhaps situated behind an ear. The needle-like ports permit penetration into the retina, securing the device and locating glutamate release close to its cellular targets. Glutamate release is envisaged to be controlled by a photoinduced electric field of an integrated organic photodiode (OPD) formed around each microneedle inducing electroosmotic actuation. **(B)** Epiretinal placement of the needle array. **(C)** Subretinal placement of the needle array.

Figure 1 shows the components of a chemical retinal prosthesis. Two sites for implantation of retinal prostheses have been used for electrical stimulation and both have been investigated as potential sites for placement of chemical stimulation arrays. In one case, so-called epiretinal placement, a stimulation array is inserted into the vitreal chamber so that the points of stimulation lie adjacent to the retina’s inner surface, close to the retinal ganglion cells (Figure 1B). The second potential site for array placement is in the space usually occupied by, now degenerated, photoreceptors. This is referred to as subretinal and results in the stimulation points adjacent to bipolar and horizontal cells (Figure 1C). The expected advantage of subretinal placement is that it would provide a naturalistic location from which glutamate could interact with OFF and ON bipolar cells, leading to natural modulation of the retina’s OFF and ON pathways. The advantage of epiretinal placement would be that the vitreal space affords the possibility of a larger intraocular well for glutamate.

The surgical approaches taken for the implantation of electrical retinal prostheses are similar to those that would apply for the implantation of a chemical retinal prosthesis. For subretinal implantation, a temporary retinal detachment is involved, and methods are taken to secure the implant for both epiretinal and subretinal placements. For a fuller summary of surgical details for subretinal implants (see Besch et al., 2007; MacLaren, 2017) and for epiretinal implants see (Humayun et al., 2012).

All retinal prostheses require a camera system to capture an image and a means to translate that image into a pattern of retinal ganglion cell stimulation. Electrical prostheses accomplish this through pixelated electrical stimulation. Chemical retinal prostheses would use pixelated release of neurotransmitter. In this case, the spacing of pixels and its resultant spatial resolution are limited by the potential activation field of transmitter spread from an injection port (pixel). The potential for spatial resolution equivalent to or superior to that achieved for electrical stimulation has been demonstrated for both epiretinal (Inayat et al., 2015) and subretinal chemical stimulation (Rountree et al., 2016, 2018). Nevertheless, further advance is needed to reach the visual acuity of a person defined sighted legally. A prototype of a chemical synapse chip was developed and shown to generate patterned retinal ganglion cell responses from *ex vivo* rat retina by Rountree et al. (2017a).

Higher spatial resolution is possible with less lateral spread of glutamate stimulation. The spread is dependent on the concentration and volume of glutamate injected. Finlayson and Iezzi (2010) investigated the concentration of glutamate needed to drive retinal responses in Sprague-Dawley and photoreceptor degenerate S334ter-4 rats when delivered to the epiretinal surface or 15-20 um below that surface. Glutamate concentrations in the range 0.4-10 mM were effective for evoking responses from retinal ganglion cells. 0.25-10 mM glutamate injected 20 um below the epiretinal surface has also been shown to be effective for stimulating-retinal ganglion cells of Hooded Long-Evans rats (Inayat et al., 2015). Inayat et al. (2015) also showed that the field of stimulation was reduced significantly when a small (< 10 pL) volume of glutamate was injected. Corresponding fields of glutamate stimulation have been reported for retinal ganglion cells of Hooded Long-Evans and photoreceptor degenerate S334ter-3 rat retinas for subretinal activation (Rountree et al., 2016, 2018, 2020). Hence, the potential for visual acuities in the legally sighted range seem attainable for a chemical retinal prothesis with either epiretinal or subretinal placement.

No functioning chemical retinal prosthesis has been implanted in an animal model to date. But progress toward the fabrication of an implantable device has been made. Figure 1A shows what is envisaged. Rountree et al. (2020) showed that subretinal stimulation of retinal ganglion cells is improved substantially when injection ports penetrate some distance into the retina from the subretinal surface. The same is likely true for epiretinal stimulation, so a stimulation array with needle-like injection ports is envisaged. While most demonstrations of glutamate activation of retinal ganglion cells from the epiretinal (Finlayson and Iezzi, 2010; Inayat et al., 2015) or from the subretinal side (Rountree et al., 2016, 2018, 2020) have relied on pneumatic injection (Rountree et al., 2017b), it was recognized by Peterman et al. (2004) that electroosmotic flow might be better suited as a neurotransmitter release mechanism in a microfabricated device. Kare et al. (2021) have shown recently that electroosmosis can be used effectively to dispense glutamate to rat retinas and evoke responses from its ganglion cells with properties similar to those evoked through pneumatic actuation.

Silicon is not a suitable microfabrication material for an implantable retinal prosthesis because of its mechanical rigidity. Peterman et al. (2003) investigated the use of PDMS/SU-8, which, as a softer material, would more easily conform to the curvature of a retina. They showed its application as such for an epiretinal implant in a New Zealand White rabbit. SU-8 has been used widely as a structural material for applications such as complex microfluidic devices, lab-on-a-chip systems, and biomedical implants (Sato et al., 2006; Chaudhri et al., 2010; Altuna et al., 2013). Unique and novel fabrication techniques like grayscale lithography, e-beam lithography, X-ray lithography, and holographic lithography can be used to generate sophisticated 3-D and ultra-high aspect ratio SU-8 microstructures with few processing steps (Bogdanov and Peredkov, 2000; Bilenberg et al., 2006; Rammohan et al., 2011; Lee et al., 2015). Bogdanov and Peredkov (2000) fabricated 100:1 aspect ratio SU-8 microstructures using X-ray lithography. Bilenberg et al. (2006) fabricated ∼24 nm SU-8 nanochannels using e-beam lithography, and Rammohan et al. (2011) fabricated 3-D SU-8 microfluidic devices in a single step using grayscale lithography. So, the capacity to build small microfluidic systems with SU-8 has been well-established.

As demonstrated by Peterman et al. (2003), the mechanical flexibility of SU-8 is an advantage in fabricating biomedical implants. Tijero et al. (2009) developed flexible implantable SU-8 microprobes to monitor ischemia in rat kidneys. The development of flexible probes was necessary to minimize the loss of functionality due to micromotion between probe and tissue. The mechanical and electrical properties of SU-8 probes outperformed standard silicon and silicon carbide probes due to their flexibility. The processing costs and times were also lower for the development of SU-8 probes compared to silicon and silicon carbide probes. Huang et al. (2014) developed SU-8 neuroprobes for electrophysiological recordings and characterized the mechanical flexibility and biocompatibility both *in vitro* and *in vivo*. They concluded that the SU-8 neuroprobes not only withstood the resistance from brain tissue during surgical implantation but also possessed enough flexibility to prevent chronic tissue damage. Apart from SU-8 microprobes, flexible 3-D SU-8 microfluidic devices have also been developed using lamination technology (Abgrall et al., 2006).

One potential area of concern for the design of a chemical retinal prosthesis is the space occupied by the reservoir for glutamate. However, preliminary calculations made by Inayat et al. (2015) suggest that an intraocular glutamate well (Figure 1A) with capacity for multi-day use would be possible, especially if located in the vitreal chamber. Moreover, whether glutamate is pooled for a subretinal or for an epiretinal prosthesis, replenishment of its well on a daily basis should work. Figure 1A envisages an external reservoir that would permit long-term glutamate dispensing and easy replenishment of the intraocular well.

## Biocompatibility

The *in vitro* and *in vivo* biocompatibility of SU-8 have been thoroughly investigated. Kotzar et al. (2002) demonstrated the biocompatibility of SU-8 using cell culture models. Nemani et al. (2013) performed detailed *in vitro* and *in vivo* testing of the biocompatibility of SU-8 material. Cytotoxic tests, hemolytic assays, and agar diffusion assays have all demonstrated cell viability *in vitro*. SU-8 pads were implanted subcutaneously in mice for *in vivo* assessment of SU-8 biocompatibility. Based on these tests, the biocompatibility of SU-8 compares well to FDA approved implantable materials like silicone and medical steel. Márton et al. (2020) assessed the biocompatibility of SU-8 devices implanted in the neocortex of rats. Based on their observations, SU-8 is a suitable implantable material for central nervous system tissue. Taken together, ease of fabrication, chemical stability, mechanical flexibility, and biocompatibility, SU-8 would appear to be a suitable material with which to fabricate a chemical retinal prosthesis.

## Stimulation of Retinal Ganglion Cells With Glutamate

Glutamatergic stimulation of retinal ganglion cells has been accomplished to date through localized pulsatile dosing of the retina and this is how delivery of the neurotransmitter is envisaged for a chemical retinal prosthesis. This differs from the natural pattern of glutamate release by photoreceptors where the transmitter is released constantly in darkness and reduced by light. However, pulsatile release has been shown to evoke generally naturalistic spiking of retinal ganglion cells and has the benefit of limiting the quantity present in the retina of a potentially excitotoxic chemical.

The results of Finlayson and Iezzi (2010) indicated that epiretinal stimulation through glutamate often creates an initial suppression of retinal ganglion cell firing followed by excitation. However, for other retinal ganglion cells, glutamate stimulation was purely excitatory. Inayat et al. (2015) also report a complex mix of excitatory and inhibitory glutamate induced retinal ganglion cell responses with epiretinal stimulation. It seems likely that the inhibitory responses result from glutamate drive to amacrine cells which in turn provide GABAergic or glycinergic inhibitory drive to ganglion cells. The natural response of some retinal ganglion cells to visual stimulation is suppression of firing (Rodieck, 1967; Troy et al., 1989) so it would be incorrect to conclude that an inhibitory response from a ganglion cell to glutamate must imply inappropriate stimulation of the retinal output through glutamate. Nevertheless, the complex pattern of responses of retinal ganglion cells to glutamate and the fact that, although less than the roughly forty types found in mouse (Baden et al., 2016; Rheaume et al., 2018), there are likely twenty or more types of ganglion cell in the human retina (Masri et al., 2019; Peng et al., 2019; Yan et al., 2020; Kim et al., 2022) throws into doubt the likelihood of evoking a fully naturalistic pattern of retinal ganglion cell activation from glutamate delivered through epiretinal placement.

## Naturalistic Stimulation

**FIGURE 2:**
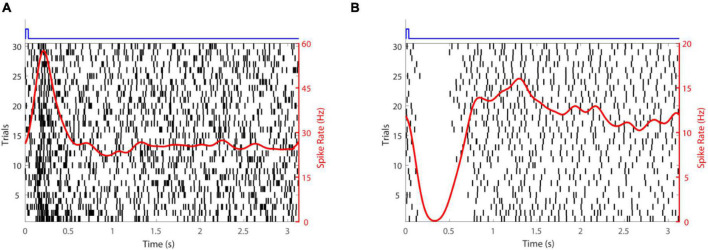
Retinal ganglion cell responses to glutamate stimulation. **(A)** An excitatory response. **(B)** An inhibitory response. The time-course of glutamate delivery is indicated by the blue stimulus traces shown at the top. Thirty trials (left axis) are shown for each cell. Action potentials discharged are indicated by vertical lines and the spike rate (right axis) averaged across trials (a smoothed peri-stimulus time histogram) is given by the red line.

**FIGURE 3:**
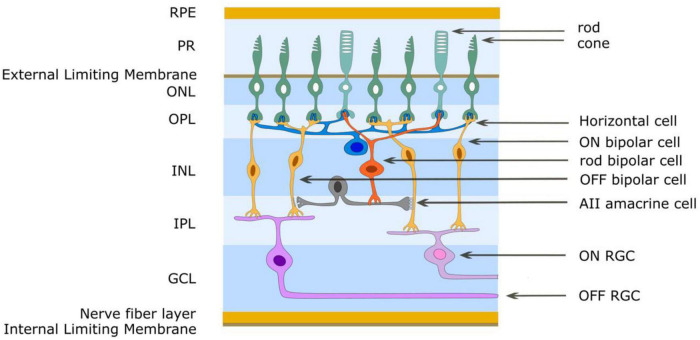
Simplified schematic diagram of the OFF and ON retinal pathways. Glutamate is released from photoreceptor terminals in darkness in the outer plexiform layer and generates the OFF pathway by exciting OFF bipolar cells through ionotropic glutamate receptors. Glutamate acts on mGluR6 receptors of ON bipolar cells to create sign-inverting inhibitory responses, thus creating the ON pathway. Light reduces glutamate release from photoreceptors. The AII amacrine cell acts to create the OFF and ON pathways for rod-driven (scotopic) vision. The rod bipolar cell has mGluR6 receptors and gives an ON response to light. It also uses glutamate as its neurotransmitter and through ionotropic glutamate receptors depolarizes AII amacrine cells. This cell provides differential output to cone bipolar cells in the inner plexiform layer. ON cone bipolar cells are connected to the AII amacrine cell through gap junctions (excitatory) while OFF cone bipolar cells are connected to the AII amacrine cell by glycinergic (inhibitory) chemical synapses. RPE retinal pigment epithelium, PR photoreceptor outer segments, ONL outer nuclear layer, OPL outer plexiform layer, INL inner nuclear layer, IPL inner plexiform layer, GCL ganglion cell layer.

Subretinal placement would seem more likely to evoke a natural retinal response since the stimulation array seeks to replace photoreceptors and engage retinal circuits normally. Figure 2 shows responses of two retinal ganglion cells to injection of glutamate subretinally. Given that glutamate excites OFF bipolar cells and inhibits ON bipolar cells (Figure 3), it was somewhat surprising that Rountree et al. (2016) found that the discharges of retinal ganglion cells in Hooded Sprague-Dawley rats showed an unexpected mix of excitatory and inhibitory responses to subretinal application of glutamate like that seen with epiretinal stimulation. The discharges of OFF retinal ganglion cells were depressed more often than those of ON or of ON-OFF retinal ganglion cells, so there was differential stimulation of the OFF and ON pathways but in a complex manner. It seems likely that the glutamate acted at its receptors on bipolar, amacrine and ganglion cells. Measurements of retinal local field potentials point to glutamate evoking responses in neurons of the inner nuclear layer (i.e., amacrine and bipolar cells). A similar pattern of subretinal differential (excitatory and inhibitory) stimulation of retinal ganglion cells has also been shown to occur in photoreceptor degenerate S334ter-3 rats. Preliminary investigation of subretinal stimulation with glutamate has also provided evidence of network-mediated responses in the photoreceptor degenerate rd1/rd1 mouse (Haq et al., 2018). Further investigation of how retinal circuits are engaged both from epiretinal and subretinal sides is needed nevertheless for one to design a chemical prosthesis that could best mimic the retina’s natural output.

**FIGURE 4:**
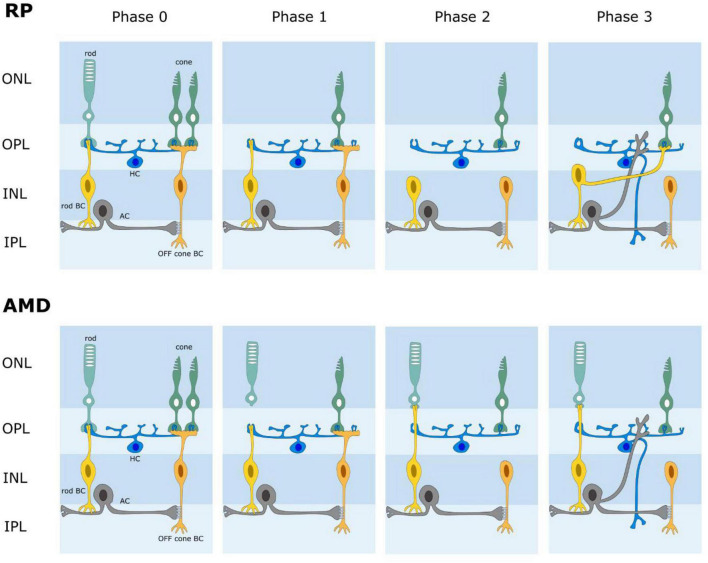
Schematic diagram of retinal wiring stages following photoreceptor degeneration. AMD age-related macular degeneration. RP retinitis pigmentosa. In both AMD and RP, the retina undergoes a phased loss of cells and rewiring of its circuits. Phase 0 is normal retina. Phases 1-3 illustrate some changes that have been reported; i.e., photoreceptor loss, loss of connections between photoreceptors and bipolar cells, novel connections between rods and cone bipolar cells, retinal rewiring.

The retina is known to reorganize its circuits after photoreceptor degeneration (Figure 4). Rodent models of retinal degeneration have been studied extensively (Strettoi and Pignatelli, 2000; Strettoi et al., 2002, 2003; Marc and Jones, 2003; Marc et al., 2007, 2008) and we also know something about how human retinas reform connections following photoreceptor loss. The animal models demonstrate that retinal degeneration passes through three stages: (1) neuronal death, (2) cell migration, and (3) circuit rewiring. Animals with cone-decimating degeneration lose their rods and cones during phase 1. In phase 2, the bipolar cells retract their dendrites and lose kainate (OFF bipolars) and mGluR6 (ON bipolars) responsivity; i.e., they become unresponsive to glutamate (but see Gayet-Primo and Puthussery, 2015). During phase 3, surviving bipolar, horizontal and amacrine cells generate anomalous sprouts which create novel synaptic connections with each other, thus rewiring retinal circuits. For animals with cone-sparing degeneration, cones outlive rods during phase 1. Some rod bipolar cells then create ectopic synapses with cones and transition from mGluR6 (ON) signaling to kainate/AMPA (OFF) signaling. As in cone-decimating degeneration, bipolar dendrites became atrophic during phase 2. But, sprouting from bipolar cells and synaptic rewiring in phase 3 is slowed because some rod bipolar cells remain in contact with cone pedicles. While studies of changes in photoreceptor degenerate human retina have been less extensive, the same general observations seem to hold (Sullivan et al., 2007; Jones et al., 2012, 2016; Pfeiffer et al., 2020). While one might be inclined to believe that the inner retina is unaffected, cell migration of some retinal ganglion cells has been reported. One would be wise therefore to assume that post-photoreceptors retinal tissue has some significant differences from the physiological retina. Until a full characterization of the changes that occur for humans with photoreceptor degeneration and a means to define them for individual patients has been attained, it makes little sense to seek perfection in the design of a retinal prosthesis. The sound approach is to seek to drive retinal output as close to nature as possible and trust that higher levels of the visual system can learn to make sense of the signals generated. The fact that the patterns of glutamate activation in intact and photoreceptor degenerate rat models are seemingly similar offers hope that a chemical retinal prosthesis will function well for patients with photoreceptor loss.

One additional potential advantage of a chemical retinal prosthesis in the context of retinal remodeling following photoreceptor degeneration is that release of glutamate from the subretinal side might slow or counteract the processes driving remodeling. It would also be possible to corelease other chemical agents (e.g., trophic factors) that could guide the remodeling in a functional beneficent manner.

## Temporal Resolution

Although this remains to be demonstrated with a retinal prosthesis, electrical stimulation of neurons is likely limited by the refractoriness of their discharges. The ability to control the temporal frequency of chemical neural stimulation has more physical limitations. It takes time to dispense glutamate to its targets and time for those targets to respond. Early work by Finlayson and Iezzi (2010) suggested that activation rates with glutamate up to 3-5 Hz are possible. More recent work indicates that the range may be somewhat broader than this but still well below the critical flicker fusion frequency of photopic vision. Chemical retinal prostheses should have the temporal resolution to support visual tasks like reading but not those that require detection of rapid motion.

## Excitotoxicity

One of the main concerns for a prosthesis that dispenses glutamate to the retina is the known excitotoxicity of this chemical (Izumi et al., 1999; Delyfer et al., 2005; Kritis et al., 2015). Excessive stimulation of glutamate receptors is known to cause cell death. The Müller cells of normal retina uptake excess glutamate and convert it into non-toxic glutamine via glutamine synthetase (GS), then the glutamatergic neurons uptake the glutamine to synthesize glutamate (Riepe and Norenberg, 1978; Ishikawa, 2013; Liu et al., 2013). This glutamate-glutamine cycle allows neurons to avoid glutamate excitotoxicity. Inayat et al. (2015) estimated that the clearance rate for glutamate in a normal retina would be sufficient to avoid glutamate toxicity with a chemical retinal prosthesis. But, this estimate did not consider changes to the action of Müller cells in photoreceptor degenerate retina.

The normal retina expresses a low concentration of glutamate and high concentrations of glutamine and taurine in its Müller cells (Marc et al., 1995). At an early-stage of retinal degeneration, even before the initiation of photoreceptor loss, changes to this pattern of expression occur (Fletcher and Kalloniatis, 1996, 1997; Fletcher, 2000; Gibson et al., 2013; Pfeiffer et al., 2016). The immunoreactivities of glutamate and glutamine are both elevated in Müller cells, implying anomalous glutamate degradation. Hence, whether glutamate dispensed from a chemical retinal prosthesis would be excitotoxic remains an open question needing further investigation.

## Next Steps

The next step in development of a chemical retinal prosthesis is to fabricate a microdevice that could be implanted in an animal model and test whether it can generate visual responses in cortical neurons (Frankowski et al., 2021) and behaviors indicative of visual perception (Feng et al., 2016). In this article, we have provided details of the materials that could be used for fabrication of such a device and a mechanism (electroosmosis) suitable for glutamate release. Additional investigation of how retinal neural circuits are modified in photoreceptor degeneration and how these modifications might affect glutamate driven retinal responses would help craft a prosthesis that maximizes the potential for visual restoration. *In vivo* testing of glutamate toxicity for an implanted device is needed too. We are entering an exciting period for chemical retinal prosthesis development. After a long period of preliminary work, establishing feasibility and investigating the properties of retinal stimulation, the materials and mechanisms for microfabrication, the pieces seem now to be in place to build and test the first implantable device.

## Author Contributions

JW and JT conceived the idea and drafted the manuscript. PR, S-SK, CR, and JF made important contributions. JW and CR created the figures. JT led the overall work. All authors read and approved the submitted version.

## Conflict of Interest

The authors declare that the research was conducted in the absence of any commercial or financial relationships that could be construed as a potential conflict of interest.

## Publisher’s Note

All claims expressed in this article are solely those of the authors and do not necessarily represent those of their affiliated organizations, or those of the publisher, the editors and the reviewers. Any product that may be evaluated in this article, or claim that may be made by its manufacturer, is not guaranteed or endorsed by the publisher.
